# Fecal microbial and metabolic characteristics of swine from birth to market

**DOI:** 10.3389/fmicb.2023.1191392

**Published:** 2023-09-18

**Authors:** Huan He, Mingzhi Yang, Wentao Li, Zeqing Lu, Yizhen Wang, Mingliang Jin

**Affiliations:** ^1^Key Laboratory of Molecular Animal Nutrition, Ministry of Education, Hangzhou, Zhejiang, China; ^2^Key Laboratory of Animal Nutrition and Feed Science (Eastern of China), Ministry of Agriculture and Rural Affairs, Hangzhou, Zhejiang, China; ^3^National Engineering Laboratory for Feed Safety and Pollution Prevention and Controlling, Zhejiang University, Hangzhou, Zhejiang, China; ^4^Key Laboratory of Animal Feed and Nutrition of Zhejiang Province, Hangzhou, Zhejiang, China; ^5^College of Animal Sciences, Institute of Feed Science, Zhejiang University, Hangzhou, Zhejiang, China

**Keywords:** fecal microbiome, microbial metabolome, dynamic change, from birth to market, swine

## Abstract

**Introduction:**

Recently, the research on pig intestinal microbiota has become a hot topic in the field of animal husbandry. There are few articles describing the dynamic changes of porcine fecal microbiota and metabolites at different time points from birth to market.

**Methods:**

In the present study, 381 fecal samples were collected from 633 commercial pigs at 7 time points, including the 1st day, the 10th day, the 25th day, the 45th day, the 70th day, the 120th day, and the 180th day after the birth of swine, were used for microbiome analysis by Illumina MiSeq sequencing methods while 131 fecal samples from 3 time points, the 10th day, the 25th day, and 70th day after birth, were used for metabolome analysis by LC–MS methods.

**Results:**

For the microbiome analysis, the fecal microbial richness increased over time from day 1 to 180 and the β-diversity of fecal microbiota was separated significantly at different time points. Firmicutes were the main phyla from day 10 to 180, followed by Bacteroides. The abundance of *Lactobacillus* increased significantly on day 120 compared with the previous 4 time points. From day 120 to day 180, the main porcine fecal microbes were *Lactobacillus*, *Clostridium_sensu_stricto_1*, *Terrisporobacter* and *Streptococcus*. *Clostridium_sensu_stricto_1* and *Terrisporobacter* increased over time, while *Lactobacillus*, *Escherichia-Shigella, Lachnoclostridium* decreased with the time according to the heatmap, which showed the increase or decrease in microbial abundance over time. For the metabolome analysis, the PLS-DA plot could clearly distinguish porcine fecal metabolites on day 10, 25, and 70. The most different metabolic pathways of the 3 time points were Tryptophan metabolism, Sphingolipid signaling pathway, Protein digestion and absorption. Some metabolites increased significantly over time, such as Sucrose, L-Arginine, Indole, 2,3-Pyridinedicarboxylic acid and so on, while D-Maltose, L-2-Aminoadipic acid, 2,6-diaminohexanoic acid, L-Proline were opposite. The correlation between fecal metabolites and microbiota revealed that the microbes with an increasing trend were positively correlated with the metabolites affecting the tryptophan metabolic pathway from the overall trend, while the microbes with a decreasing trend were opposite. In addition, the microbes with an increasing trend were negatively correlated with the metabolites affecting the lysine pathway.

**Discussion:**

In conclusion, this study elucidated the dynamic changes of porcine fecal microbiota and metabolites at different stages from birth to market, which may provide a reference for a comprehensive understanding of the intestinal health status of pigs at different growth stages.

## Introduction

The gut microbiota of pigs has received a great deal of attention in recent years, as pigs are not only an important source of animal-derived food, but also an excellent biomedical model of human health (Li et al., 2020; Lunney et al., 2021). Microbes have essential functions for host health, such as nutritional supplementation, immune regulation, disease prevention, and physical development (De Almeida et al., 2019; Čoklo et al., 2020; Barrea et al., 2021; Zhou et al., 2021). The porcine gut microbiome has been reported to be associated with feed efficiency, fat deposition and growth performance (McCormack et al., 2019; Bergamaschi et al., 2020; Jiang et al., 2021).

Currently, great progress has been made in understanding the swine gut microbiome. The porcine gut microbiome associated with different stages may be determined by differences in diet and gut physiology at different growth stages (Wang et al., 2019a,b; Li et al., 2020). Our previous study investigated the characteristics of the fecal microbiota in 120 pigs of different breeds, growth stages and genders, and finally found that the growth stage was the largest contributor to the pig gut microbiota (Wang et al., 2022). In addition, another researcher combined microbiome and metabolome strategy to evaluate the effects of probiotics or symbiotic addition to sows’ diets on colonic microbiota and their metabolites in offspring (Zhu et al., 2022). Although progress has been made in understanding the swine gut microbiome, there is still a lack of systematic analysis that combine microbiome and metabolome to dissect the changes of fecal microbiome and metabolome of pigs from birth to market.

In the present study, we investigated the changes of fecal microbiota and their metabolites at different growth stages of pigs, hoping to take some measures to regulate pig gut microbes to enhance pig gut health and improve pig growth performance in the near future. Fecal samples collected from 633 commercial pigs (Duroc × Landrace × Yorkshire crossbred pigs) were randomly selected, of which 381 samples (7 time points: day 1, 10, 25, 45, 70, 120, and 180 after birth) were subjected to microbiome analysis with Illumina MiSeq sequencing methods, while 131 samples (3 time points: day 10, 25, and 70 after birth) were subjected to metabolome analysis with LC–MS methods, which aimed to illustrate the following three key points: (i) the swine fecal microbiota from birth to market was characterized; (ii) the pan and core fecal microbiota and predicted metagenomic functions were investigated; and (iii) the fecal metabolite changes and their association with microbes were investigated.

## Results

### Data assessment

Here, the data of 381 microbial samples and 131 metabolic samples were analyzed. In terms of microbial data analysis, fecal samples from seven time points on day 1, 10, 25, 45, 70, 120, and day 180 with the number of 57, 70, 72, 24, 58, 50, and 50 were use, respectively. A total of 20,738,913 sequences were obtained and the average length of each sample was 416.17 ± 23.43. For metabolic data analysis, fecal samples from three time points on day 10, 25, and 70 with the number of 25, 60, and 46 were used, respectively. In pos ion mode, a total of 447 effective peaks were obtained, of which 312 metabolites in Library (such as HMDB and Lipid maps) and 151 metabolites in KEGG. As for neg ion mode, a total of 395 effective peaks were obtained, of which 309 metabolites in Library (such as HMDB and Lipidmaps) and 104 metabolites in KEGG.

### Swine fecal microbial communities (α-diversity and β-diversity)

**Figure 1:**
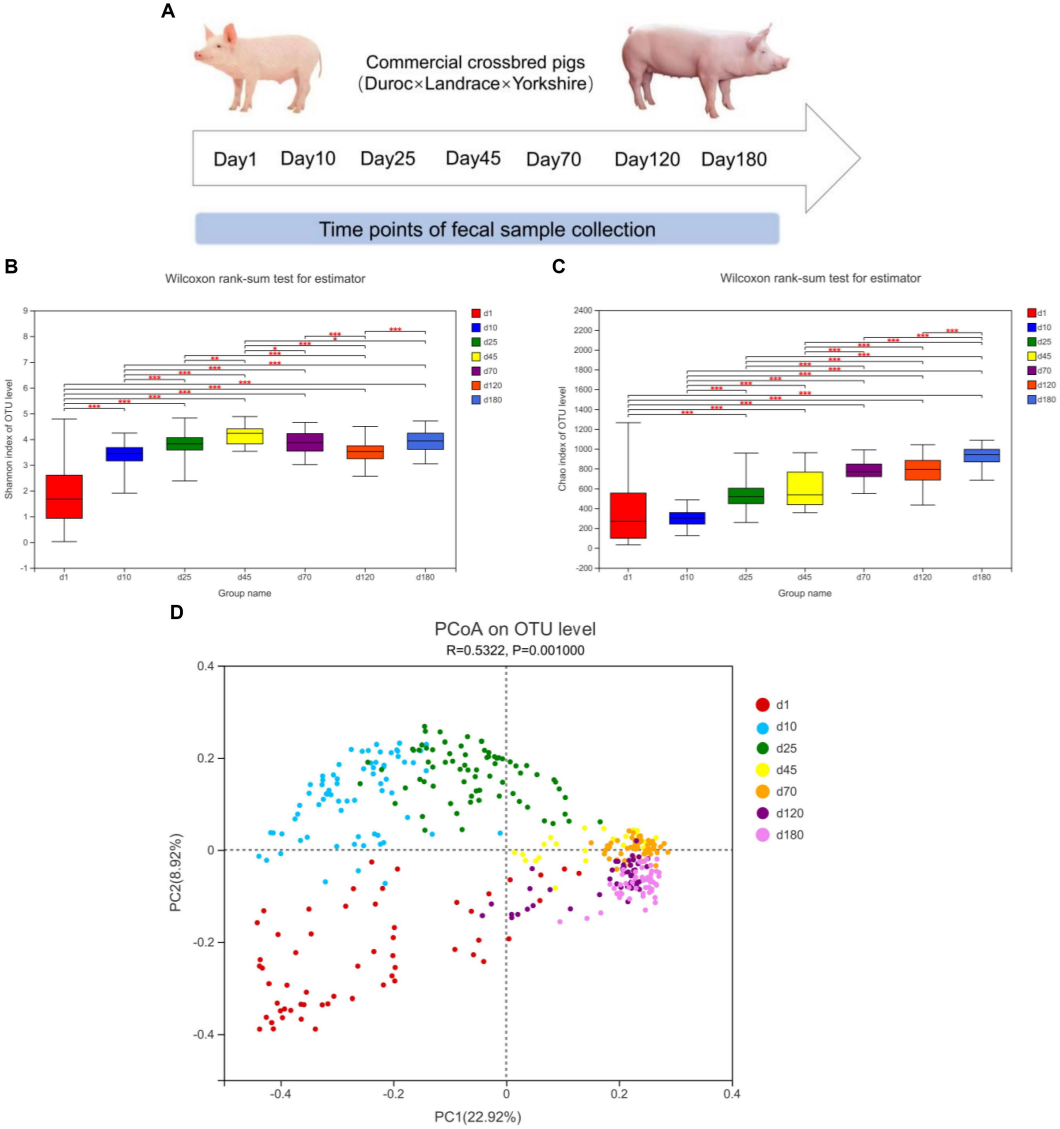
Experimental design and description of swine intestinal microbial communities. **(A)** Experimental design and sampling time points. **(B,C)** α-diversity (Shannon and Chao index), *means *p* < 0.05, **means *p* < 0.01, ***means *p* < 0.001. **(D)** PCoA plot of β-diversity based on unweighted unifrac distance.

The α-diversity (Shannon and Chao index) and β-diversity of fecal microbiota of different time points from birth to market were shown in Figure 1. As shown in Figure 1B, from day 1 to 45, the diversity of fecal microbes increased significantly over time on the general trend. From day 1 to 180, the richness of fecal microbes also increased significantly over time on the general trend (Figure 1C).

We further investigated the shifts of bacterial taxa from birth to market, including suckling, nursery and finishing stages. Principal coordinates analysis (PCoA) based on unweighted unifrac distance revealed the clustering of microbiota composition at the seven time points (Figure 1D). The closer the distance in the figure, the closer the composition of the fecal microbiota was. From the observation of PCoA, the distances between day 1, 10, and 25 were farther, while the distances between day 45, 70, 120, and day 180 were closer, implying that the composition of the fecal microbiome was relatively stable from day 45.

### Microbial composition and the linear discriminant analysis effect size analysis of different microbiota in swine intestine

**Figure 2:**
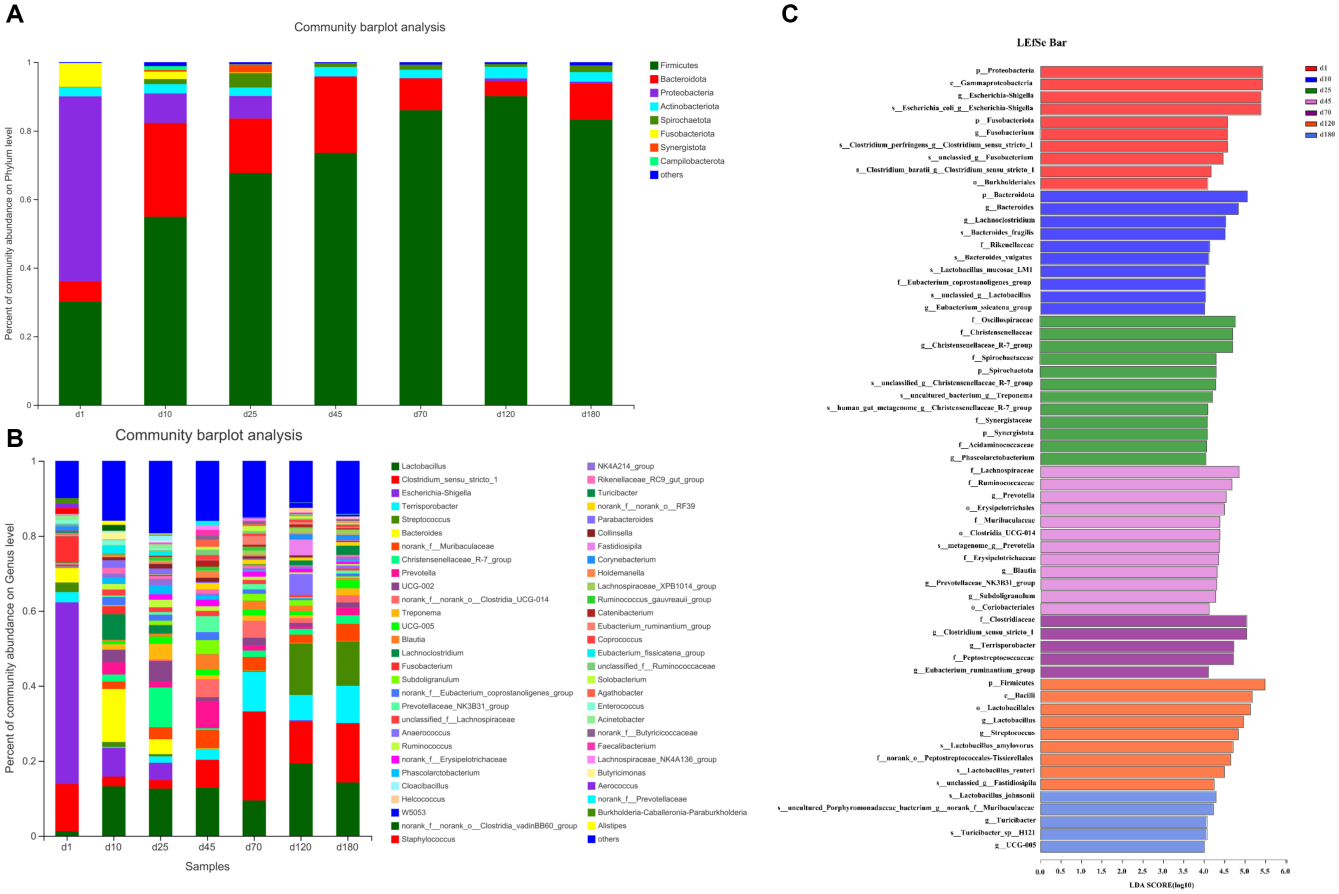
The overall microbial composition at phylum and genus level. **(A)** The phylum-level abundance. **(B)** The genus-level abundance. **(C)** LEfSe plots of different microbiota in porcine feces based on OUT level (LDA > 4).

The overall microbial composition at phylum (Figure 2A) and genus (Figure 2B) level was presented. As shown in the Figure 2A and Supplementary Figure S1A, from day 1 to 120, the abundance of Firmicutes in the porcine feces increased gradually and significantly. Firmicutes were the main phyla from day 10 to 180, accounting for about 50–90% of all phyla, followed by Bacteroides, accounting for about 5–20% of all phyla. The relative abundance of other fecal microbes was relatively low, accounting for less than 20% on day 10 and 25, and even less than 5% from day 45 to 180. On day 10 and 25, Proteobacteria were the third most abundant phylum in relative abundance except Firmicutes and Bacteroides, while Campilobacterota was the third most abundant phylum from day 45 to 180. Differently, the relative abundance of Proteobacteria dominated on day 1, accounting for about 50%, followed by Firmicutes, accounting for about 30% and Fusobacteriota were the third most abundant phylum in relative abundance except Proteobacteria and Firmicutes.

**Figure 3:**
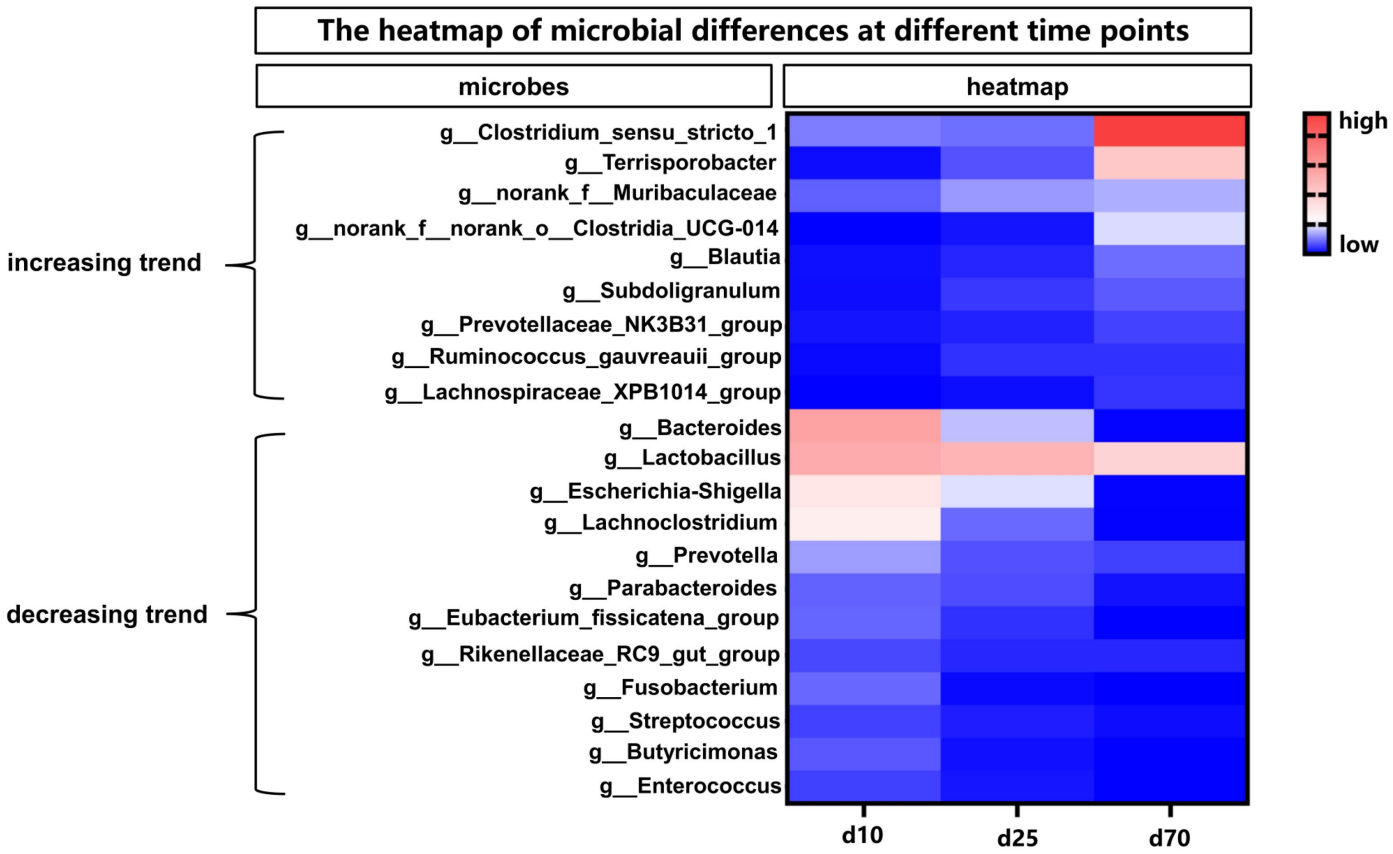
The heatmap of differential microbes at different time points. The heatmap showed the increasing or decreasing microbial abundance over time. Blue indicated low abundance and red represented high abundance.

The abundance of fecal microbiome at genus level was shown in Figure 2B and Supplementary Figure S1B. Notably, compared with day 1, the abundance of *Lactobacillus* increased significantly (*p* < 0.05) at the other 6 time points. On day 10, 25, 45, and 70, there was no significant difference in the relative abundance of *Lactobacillus*, but on day 120 there was a significant increase (*p* < 0.05) compared with these 4 time points. Curiously, the relative abundance of *Lactobacillus* on day 180 was reduced to the same level as day 10, 25, 45, and 70. Conversely, the relative abundance of *Escherichia-shigella* decreased significantly compared to day 1 at each time point starting from day 10. On the first day of birth, the fecal microbes of piglets were mainly *Escherichia-shigella*, *Clostridium_sensu_stricto_1* and *Fusobacterium*. From day 10 to 45, the genus of fecal microbiota changed dramatically, but the abundance of *Lactobacillus* remained basically unchanged between the 3 time points. From day 70 to 180, the main fecal microbes were *Lactobacillus, Clostridium_sensu_stricto_1* and *Terrisporobacter*. In addition, *Streptococcus* accounted for about 10% of the relative abundance of fecal microbes in pigs on day 120 and 180. Furthermore, the cladogram plot of LEfSe analysis was applied to find the distinct in microbial composition among different time points (Figure 2C). The results indicated that there were different enriched genera on day 1, 10, 25, 45, 70, 120, and 180. As pig grown up, high amount of *Bacteroides*, *Oscillospiraceae*, *Lachnospiraceae*, *Clostridia* and *Firmicutes* (such as *Lactobacillus amylovorus*, *Lactobacillus reuteri*, and *Lactobacillus johnsonii*) were enriched in fecal microbiota. In order to specifically analyze the pattern of change in the abundance of a particular microbe with the growth time points of pigs, we screened for a particular microbe that had a gradient pattern over time (Figure 3). As shown in Figure 3, the heatmap showed the increasing or decreasing microbial abundance over time. As the pigs grew, there were mainly 9 types of microbes such as *Clostridium_sensu_stricto_1*, *Terrisporobacter, Muribaculaceae* with an increasing trend and 12 types of microbes such as *Bacteroides, Lactobacillus, Escherichia-Shigella, Lachnoclostridium* with a decreasing trend.

### Functional predictions of swine microbiota by PICRUSt2

**Figure 4:**
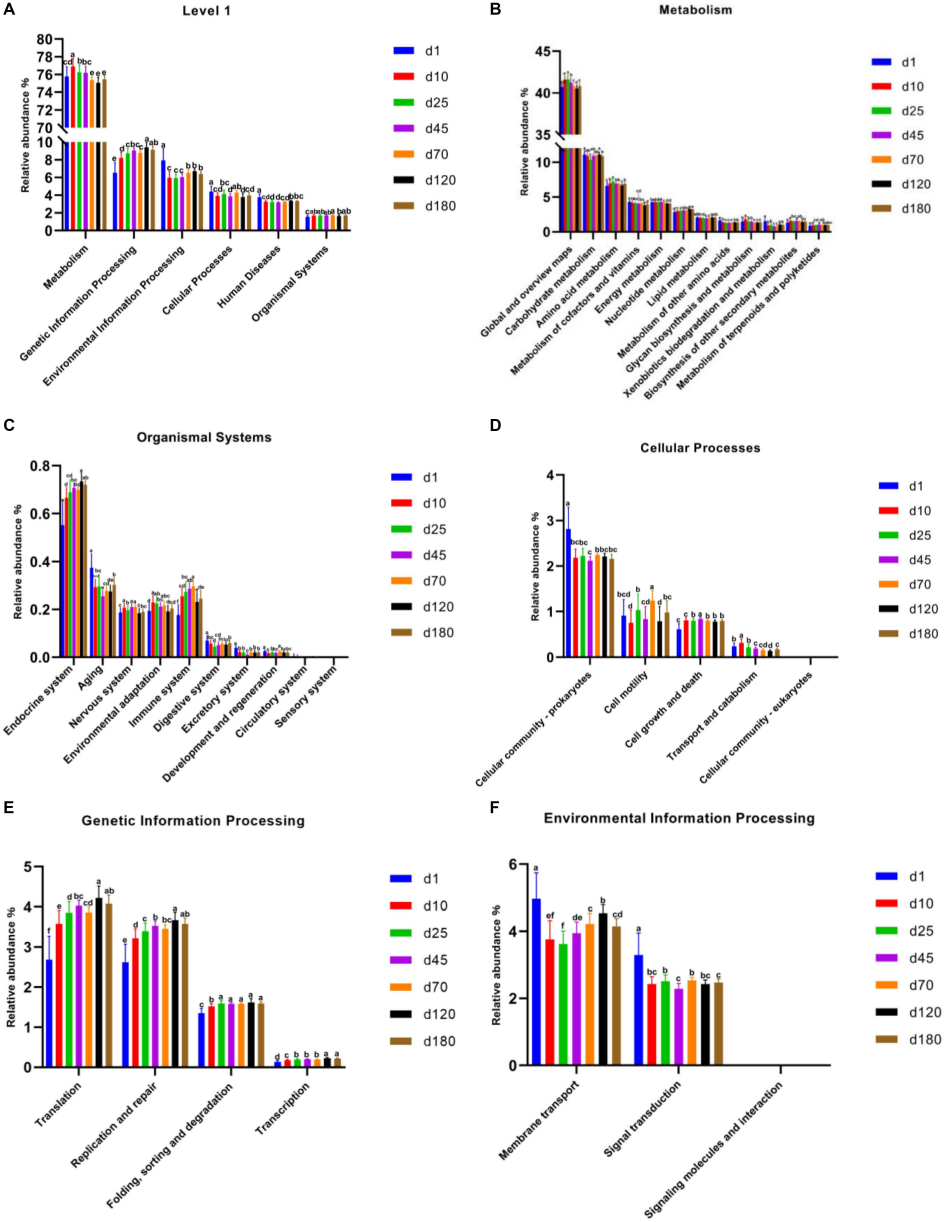
Functional predictions of swine microbiota by PICRUSt2. **(A)** General functional predictions at KEGG level 1. **(B–F)** Functional predictions KEGG level 2, including metabolism **(B)**, organismal systems **(C)**, cellular processes **(D)**, genetic information processing **(E),** and environmental information processing **(F)**.

PICRUSt2 was used for potential metabolic function prediction of the swine microbiota in this study. On the general trend, genetic information processing increased with time, and the latter time was significantly different than the former time at KEGG level 1 among most time points (Figure 4A). In detail, the time point of day 10 was significantly higher (*p* < 0.05) than day1, day 25 was significantly higher (*p* < 0.05) than day 10, day 120 was significantly higher (*p* < 0.05) than day 70 in genetic information processing. There was no significant difference in the 3 time points between day 25, 45, and 70, similar results were obtained on day 120 and 180, but they were significantly higher (*p* < 0.05) than the previous three time points. Figures 4B–F show functional prediction of KEGG level 2, including metabolism, organismal systems, cellular processes, genetic information processing and environmental information processing. Notably, cell growth and death at KEGG level 2 in metabolism significantly increased from day 10 compared to day1. In genetic information processing, translation, replication and repair and transcription increased with time, and the latter time was significantly different than the former time among most time points on the general trend. Endocrine system and immune system in organismal systems also have a similar pattern.

### Differential metabolic pathway analysis

**Figure 5:**
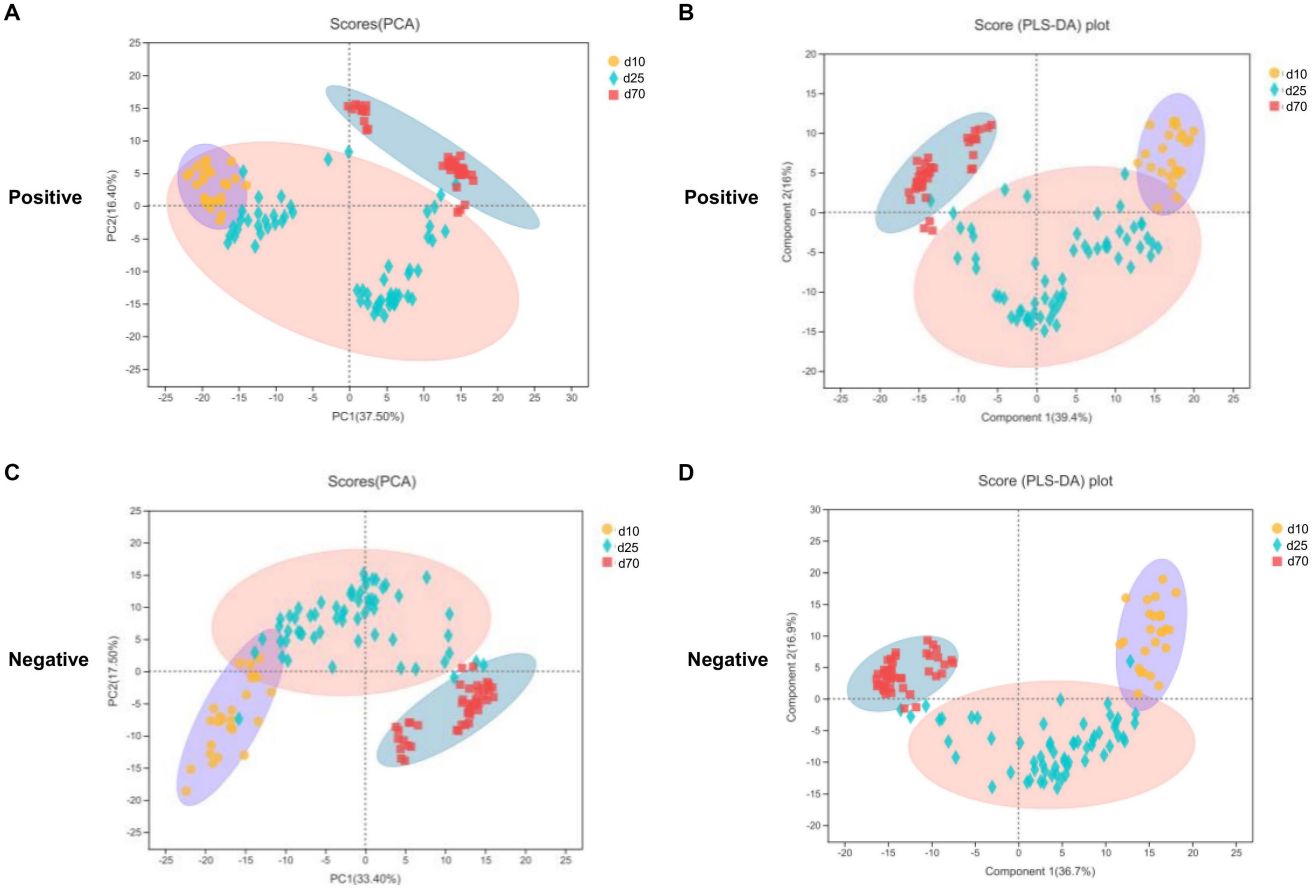
Metabolome analysis in porcine stool samples between day 10, 25, and 70. **(A,C)** PCA scores plot in the positive and negative model. **(B,D)** PLS-DA scores plot in the positive and negative model.

LC–MS was applied to investigate the changes of porcine fecal metabolites on day 10, 25, and 70. The differences of metabolomics profiles over time were shown in Figure 5. The PCA was conducted to visualize the differences in porcine fecal metabolite composition at different time points (Figures 5A,C). At the 3 time points, the metabolite composition in offspring pigs was more different in the negative model than that in the positive model (Figures 5A,C). To further verify the differences between the groups, we did an PLS-DA which clearly distinguished the metabolites (Figures 5B,D). Overall, as we could see from Figure 5, both PCA and PLS-DA scores plot could clearly distinguish porcine fecal metabolites in the positive and negative model, indicated that the fecal microbial metabolites were significantly different between day 10, 25, and 70.

**Figure 6:**
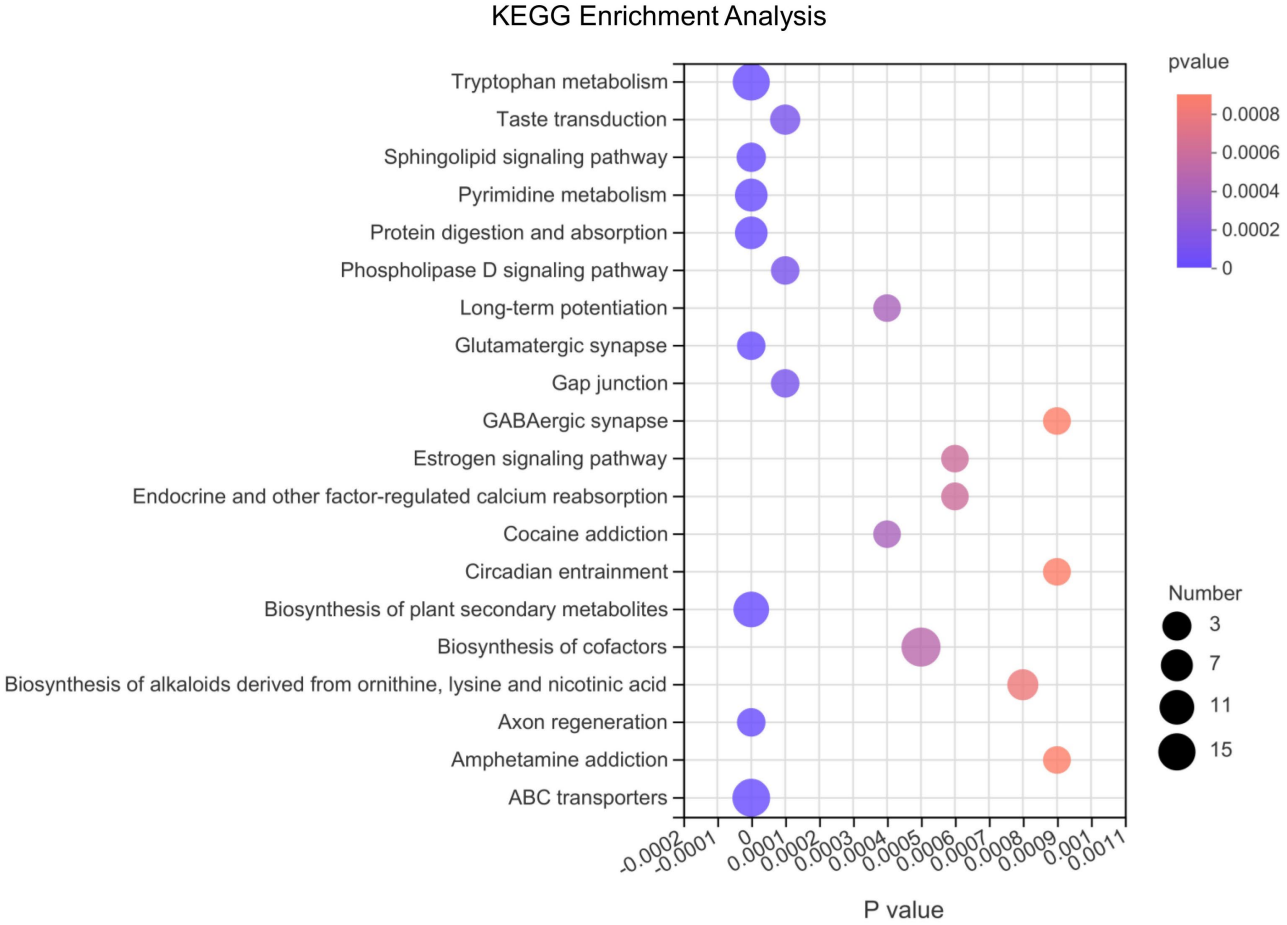
The bubble chart of enrichment analysis for metabolic pathways at 10, 25, and 70 day of age in pigs. The metabolome view shows all matched pathways according to the *p*-values from the pathway enrichment analysis and impact values from the topology analysis. The node colors varied from red to purple, indicating that the metabolites have different levels of significance. The size of the bubbles in the figure represents the number of metabolites in the pathway that are enriched in the metabolic set.

Afterwards, we conducted differential metabolite pathway analysis affected at the 3 time points as pigs grew. As shown in Figure 6, several metabolic pathways were affected by time points. The metabolome view showed all matched pathways according to the *p*-values from the pathway enrichment analysis and impact values from the topology analysis. The metabolic pathways, including Tryptophan metabolism, Sphingolipid signaling pathway, Pyrimidine metabolism, Protein digestion and absorption, Glutamatergic synapse, Biosynthesis of plant secondary metabolites, Axon regeneration and ABC transporters pathway had the most significant differences. In order of the number of metabolites in the pathway that were enriched in the metabolic set, the differential metabolic pathways were biosynthesis of cofactors, ABC transporters, tryptophan metabolism, biosynthesis of plant secondary metabolites, protein digestion and absorption, pyrimidine metabolism and so on.

### Metabolome analysis in porcine fecal metabolites of different time points

**Figure 7:**
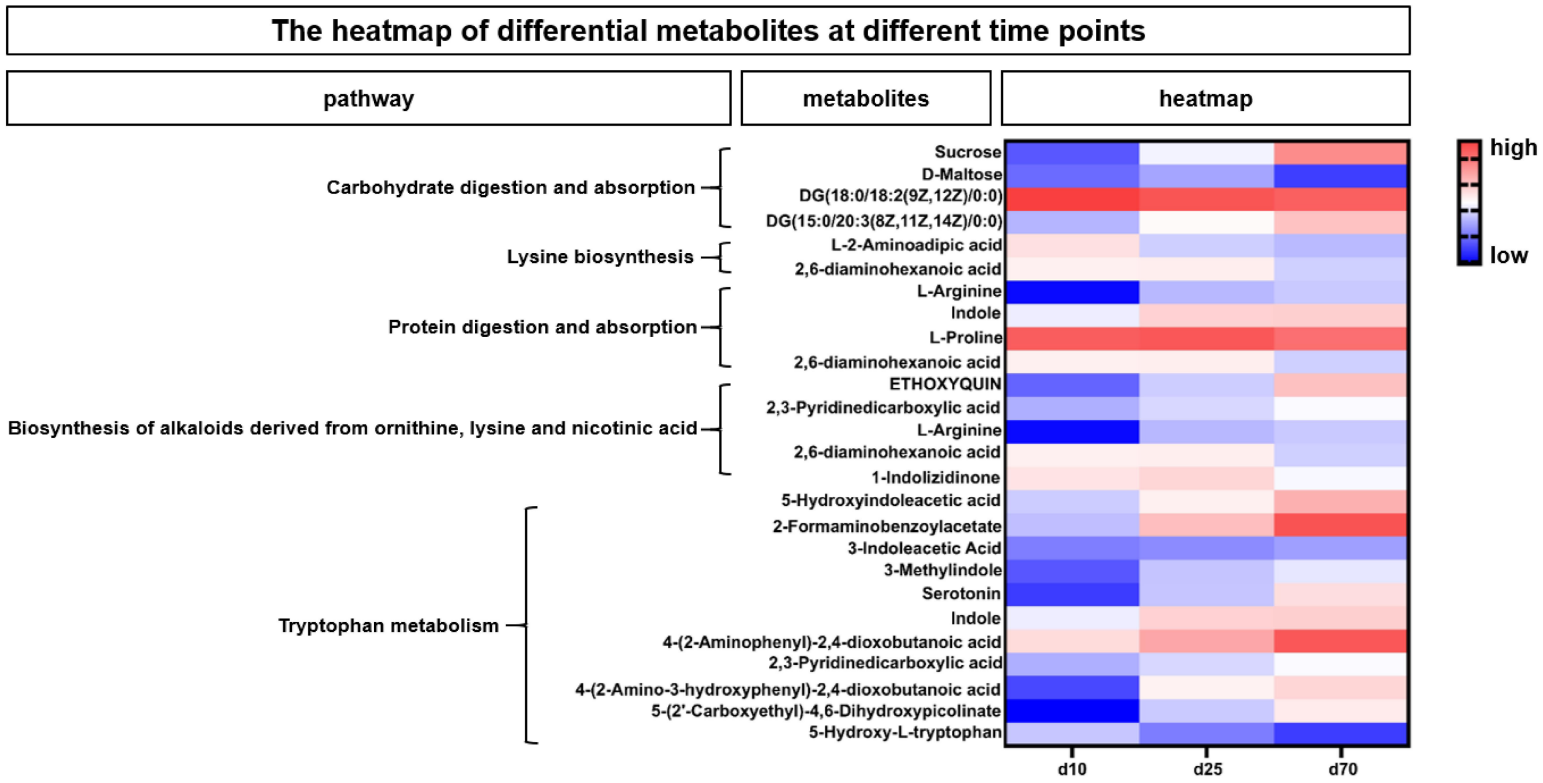
The heatmap of differential metabolites at different time points. The heatmap showed the changes of porcine fecal metabolites and their enriched metabolic pathways at different growth time points. Blue indicated low abundance and red represented high abundance.

According to the enriched metabolic pathway, we further identify the differential and pivotal metabolites affecting the pathway. As shown in Figure 7, the heatmap showed the changes of fecal microbial metabolites and their enriched metabolic pathways at different growth time points, for the differential metabolites in Carbohydrate digestion and absorption pathway, Sucrose, DG (15:0/20:3(8Z,11Z,14Z)/0:0) were increased over time, while DG (18:0/18:2(9Z,12Z)/0:0) and D-Maltose were opposite. Among the differential metabolites in Lysine biosynthesis pathway, both L-2-Aminoadipic acid and 2,6-diaminohexanoic acid were decreased over time. Among the differential metabolites affecting protein digestion and absorption pathways, two metabolites, L-arginine and indole, increased with the growth time of pigs, while the L-proline and 2,6-diaminohexanoic acid were opposite. In the biosynthesis of alkaloids derived from ornithine, lysine and nicotinic acid pathway, three metabolites, ETHOXYQUIN, 2,3-Pyridinedicarboxylic acid and L-Arginine, were significantly upregulated over time, while 2,6-diaminohexanoic acid and 1-Indolizidinone were significantly downregulated over time. Among the differential metabolites in tryptophan metabolism pathways (Figure 7), 5-Hydroxyindoleacetic acid, 2-Formaminobenzoylacetate, 3-Indoleacetic Acid, 3-Methylindole, Serotonin, Indole, 4-(2-Aminophenyl)-2,4-dioxobutanoic, 2,3-Pyridinedicarboxylic acid, 4-(2-Amino-3-hydroxyphenyl)-2,4-dioxobutanoic acid, 5-(2’-Carboxyethyl)-4,6-Dihydroxypicolinate were significantly upregulated over time, while the 5-Hydroxy-L-tryptophan was opposite.

### Correlation analysis between microbiota and metabolites

**Figure 8:**
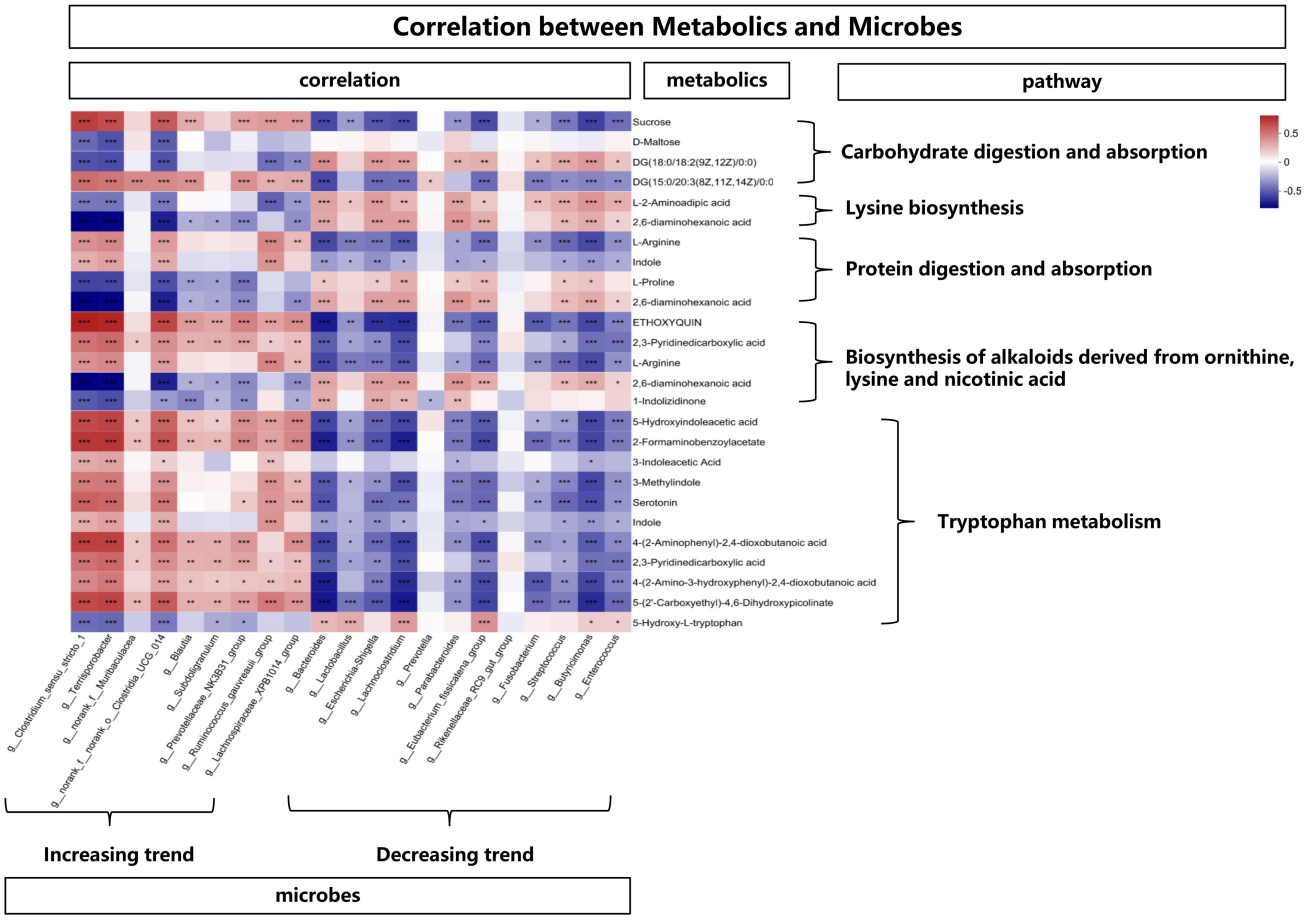
The correlation between metabolites and microbes, *means *p* < 0.05, **means *p* < 0.01, ***means *p* < 0.001.

In order to explore the functional correlation between changes of fecal microbiota and metabolites, Spearman correlation analysis was generated by calculating the Spearman correlation coefficient between microbial composition (genus level) affected by time points and metabolites. As shown in Figure 8, from the overall trend, the microbes with an increasing trend were positively correlated with the metabolites affecting the tryptophan metabolic pathway. In detail, *Clostridium_sensu_stricto_1*, *Terrisporobacter*, *Clostridia_UCG_014*, *Ruminococcus_gauvreauii_group*, *Lachnospiraceae_XPB1014_group* showed a positive correlation with 5-Hydroxyindoleacetic acid, 2-Formaminobenzoylacetate, 3-Indoleacetic Acid, 3-Methylindole, Serotonin, Indole, 4-(2-Aminophenyl)-2,4-dioxobutanoic acid, 2,3-Pyridinedicarboxylic acid, 4-(2-Amino-3-hydroxyphenyl)-2,4-dioxobutanoic acid and 5-(2’-Carboxyethyl)-4,6-Dihydroxypicolinate. While from the overall trend, the microbes with a decreasing trend were negatively correlated with the metabolites affecting the tryptophan metabolic pathway. *Bacteroides, Lactobacillus, Escherichia-Shigella, Lachnoclostridium, Parabacteroides, Eubacterium_fissicatena_group, Fusobacterium, Streptococcus, Butyricimonas, Enterococcus* showed a negative correlation with 5-Hydroxyindoleacetic acid, 2-Formaminobenzoylacetate, 3-Methylindole, Serotonin, Indole, 4-(2-Amino-3-hydroxyphenyl)-2,4-dioxobutanoic acid, 5-(2’-Carboxyethyl)-4,6-Dihydroxypicolinate. In addition, the above-mentioned microbes with an increasing trend were negatively (while the decreasing trend were positively) correlated with the metabolites (L-2-Aminoadipic acid and 2,6-diaminohexanoic acid) affecting the lysine pathway.

## Discussion

The importance of gut microbiota is widely recognized as they play a key role in animal health and their diversity provides beneficial functions for the host (Rooks and Garrett, 2016; Wiley et al., 2017; Diaz Carrasco et al., 2019; Pajarillo et al., 2021; Liu et al., 2022; Turroni et al., 2022). Although recent studies have made significant progress in our understanding of the swine gut microbiome (Wang et al., 2019a,b, 2022; Chen et al., 2021; Wang, 2022), many key ecological questions remain unanswered. Duroc × Landrace × Yorkshire crossbred pig is one of the most commonly used commercial pigs in the world because of their fast growth and high feed conversion rate (Bergamaschi et al., 2020; Wang et al., 2021). In the present study, we described the changes in the fecal microbiome and metabolome of pigs at different growth stages in order to lay a theoretical foundation for the application of probiotics.

Alpha diversity refers to the diversity within a specific area or ecosystem. Commonly used metrics include Chao, Shannon, Ace, and Simpson. In this study, we chose two indicators, Chao and Shannon, to describe the alpha diversity characteristics of pig fecal microbes. Our results indicated that as the pigs grew, the richness and diversity of the fecal microbiota increased significantly at most of the time. Guevarra et al. (2019) reported the alpha diversity index of the piglet fecal microbiota increased with age, while the variability of the microbiota between individual piglets decreased. Wang et al. (2019a,b) reported that alpha diversity, including community richness and diversity showed an overall increasing trend, which was similar to our results. Although the trends in our results and Wang’s study were not identical at some time points, the overall trend of increase was consistent.

Beta diversity analysis explores the similarity or difference of community composition between different groups of samples by comparing the species diversity between different habitats or microbial communities. Our results showed that the swine gut microbiota had significant separation on day 1, 10, and 25, but not significant on day 45, 70, 120, and 180, suggesting that the fecal microbiota of pigs tended to mature and stabilize around day 45. Our results are basically consistent with those of Wang et al. (2019a,b). Differently, our results showed that the porcine fecal microbiota had matured and stabilized from the day 45 time point, while their results showed a delay. Similarly, their results showed that the swine gut microbiota also showed a clear separation during the growing and finishing periods. Rectal swabs were collected at multiple time points prior to 6 weeks of age to study microbiota development using 16S rRNA gene analysis (Choudhury et al., 2021). Their results also showed that the fecal microbiota was also significantly separated on the 2nd, 7th, 15th, 21st, and 28th day after birth.

Our results showed that the abundance of Firmicutes in the fecal microbiota of pigs showed an overall trend of increase from birth to marketing, which were basically consistent with those of Long et al. (2022). Differently, our result showed that the Firmicutes abundance was significantly increased on day 120 compared to day 70, but their result presented that there was no significant difference in Firmicute abundance between day 60, 120, and 180. Qi et al. (2022) also reported the Firmicutes abundance gradually increased in pigs at 3, 5, and 7 months after birth. Luo et al. (2022) reported that in general, *Bacteroides, Escherichia, Clostridium, Lactobacillus, Fusobacterium*, and *Prevotella* predominate in pre-weaning piglets, followed by *Prevotella* and *Aneriacter* transition to the predominant genera, *Fusobacterium*, *Lactobacillus*, and *Astragalus* were the comparative minor genera of postweaned piglets. Strikingly, In the dynamic distribution of 10 major bacterial genera across age, the abundance of *Lactobacillus* did not increase regularly with growth, which was consistent with our results at some time points.

Changes in gut microbiota composition and abundance are accompanied by functional changes (Hu et al., 2016; Haange et al., 2019; Jiang et al., 2019; Xiao et al., 2019; Ding et al., 2021). PICRUSt2 can predict the functional composition of microbial communities in samples from amplicon sequencing results (Douglas et al., 2020; Wei et al., 2020; Li et al., 2021). Our results showed the functional prediction of level 1 and level 2 in PICRUSt2, level 1 including metabolism, genetic human diseases and organismal systems. In fact, there are few studies on the prediction of pig microbial function by PICRUSt2, let alone the prediction of pig fecal microbial function at different time points from birth to market. Beaumont et al. (2021) used PICRUSt2 to study the major differences in the functional potential of the microbiota between suckling and weaning piglets. Beaumont et al. (2021) mainly focused on two key microbial pathways involved in the degradation of dietary substrates and found the relative abundance of the predicted pathway of “fucose degradation” during lactation (day 13) was higher than that after weaning (day 23). However, PICRUSt2 had its own limitations, and the results of prediction were for reference only. Because the predictions of PICRUSt2 were based on the metabolic potential of known microbiota, but most gut microbiota was still unknown or not characterized. Researchers reported the utility of PICRUSt for inference with the default database is likely limited outside of human samples (Sun et al., 2019).

The metabolome analysis methods can dynamically display the differences in fecal microbial metabolites in pigs at different growth stages. The results of our PLS-DA analysis showed that the metabolites of the porcine fecal microbiota were clearly segregated at different growth stages of pigs. Xiong et al. (2022) also used the PLS-DA analysis method to find that the metabolites of piglets were significantly separated on the 14th, 21st, and 28th day after birth. Although they used serum rather than fecal samples, this also showed significant differences in metabolites in serum as pigs grew. We found that there are not many articles investigating porcine fecal microbial metabolites through metabolomic analysis, let alone studies at different time points and different growth stages. Hung et al. (2022) found high-fiber treatments increased two lysophosphatidylethanolamines (LysoPEs) [including lysoPE (15:0) and lysoPE (16:0)] and three lysophosphatidylcholines (LysoPCs) in grow–finish pig feces. These lysoPEs and lysoPCs are produced by hydrolyzing the sn-2 fatty acids from the respective PEs and PCs through phospholipase A2, a ubiquitous enzyme in pancreatic juice and intestinal and bacterial cells (Usui et al., 1999; Davies et al., 2014), which can all be affected by dietary fiber in pigs (Saqui-Salces et al., 2017; Ferrandis Vila et al., 2018). Two amino acids, Arg and Leu, are very important to pigs. Supplementation of Arg and Leu in pig diets was beneficial in increasing IMF deposition and improving meat color and fatty acid composition (Hu et al., 2017). Lv et al. (2012) reported that diquat-induced oxidative stress reduced growth performance increased metabolism of tryptophan via Kyn pathway that upregulated TDO mRNA expression in weaned pigs. Tryptophan, an essential amino acid, was associated with an inflammatory response when low in pig feed (Le Floc'h and Seve, 2007). Yao et al. (2011) found tryptophan is an important nutrient for nervous and immune system function, and adequate dietary intake of this amino acid is essential for growth, development, and health in animals and humans. The sphingolipid signaling pathway has been reported to be associated with fat deposition and obesity in pigs (Norred et al., 1997; Ding et al., 2019; He et al., 2020; Qiao et al., 2021). As we all know, protein is the most important and indispensable nutrient for both humans and pigs. Naturally, the pathway of protein digestion and absorption is very important for pigs (Strathe et al., 2008; Jia et al., 2021). In this study, the samples used for microbiome analysis and metabolome analysis were collected from rectal feces, not from rectal swabs, which may have been contaminated by the surrounding environment and interfered with the results.

In conclusion, this study combined microbiome and metabolome to illustrate the dynamics of pigs at different stages from birth to market. Raising a pig starts with maintaining gut health. Only when we fully understand the changes in the gut microbiota and their metabolites at different stages of pigs, can we then use human intervention to ensure the health of the porcine gut and promote the growth and production performance of pigs.

## Experimental procedures

### Animal rearing and sample collection

All procedures were carried out in accordance with the relevant provisions of the university animal experiment, and the guidelines of the Zhejiang University (Hangzhou, China) Institutional Animal Protection and Use Committee were strictly followed throughout the experiment.

The breeding experiment was carried out in the Kaihua farm of Zhejiang Tianpeng Group Co., Ltd. The experimental animals were housed and managed as follows: The sows entered the farrowing room 7 day before delivery. During the lactation stage, the temperature of the piglets’ house was jointly controlled by fans, water curtains and heat lamps. Piglets were started on creep feed on day 10 and weaned on day 26. During the nursery and finishing stage, the pigsties were allocated reasonably, and the mangers and waterers were equipped according to the density of the pigs and the temperature and ventilation of the pigs’ house. The temperature was controlled by fans and water curtains to maintain a suitable temperature. After the end of each stage of the experiment, the stiff and weak pigs were eliminated. The feed compositions for different stages of pigs were shown in Supplementary Tables S1, S2. The different feeds needs to be replaced gradually.

Stool samples from the litter pigs were obtained on day 1, 10, 25, 45, 70, 120, and 180 (381 samples in total, the number of samples were 57, 70, 72, 24, 58, 50, and 50, respectively). In addition to the rectal swabs used on the 1st day, feces were collected from fresh feces excreted by animals at other time points. The collected samples were stored in −40°C refrigerator as soon as possible. A total of 381 samples from 7 time points were used for microbiome analysis and 131 samples from 3 time points, day 10, 25, and 70 (the number of samples were 25, 60, and 46, respectively), were used for metabolome analysis.

### DNA extraction and amplicon, library construction, and 16S sequencing

The methods and steps referred to the papers of other scholars (Edgar, 2013; Chen et al., 2018; Zhang et al., 2022). Total microbial genomic DNA was extracted from fecal samples using the FastDNA^®^ SPIN Kit (MP Biomedicals Ltd., United States) according to manufacturer’s instructions. The hypervariable region V3-V4 of the bacterial 16S rRNA gene were amplified with primer pairs 338F (5′-ACTCCTACGGGAGGCAGCAG-3′) and 806R (5′-GGACTACHVGGGTWTCTAAT-3′) (Liu et al., 2016) by an ABI GeneAmp^®^ 9,700 PCR thermocycler (ABI, CA, United States). PCR amplification cycling conditions were as follows: initial denaturation at 95°C for 3 min, followed by 27 cycles of denaturing at 95°C for 30 s, annealing at 55°C for 30 s and extension at 72°Cfor 45 s, and single extension at 72°C for 10 min, and end at 4°C. PCR products were then extracted, purified and quantified. Purified amplicons were pooled in equimolar amounts and paired-end sequenced on an Illumina MiSeq PE300 platform (Illumina, San Diego, United States). The raw reads were deposited into the NCBI Sequence Read Archive (SRA) database (Accession Number: PRJNA918501). Bioinformatic analysis of the fecal microbiota was carried out using the Majorbio Cloud platform.^1^ Based on the OTUs information, alpha diversity indices including Chao1 richness and Shannon index were calculated with Mothur v1.30.1. The similarity among the microbial communities in different fecal samples was determined by principal coordinate analysis (PCoA) based on unweighted unifrac dissimilarity using Vegan v2.5–3 package. The linear discriminant analysis (LDA) effect size (LEfSe)^2^ was performed to identify the significantly abundant taxa (Phylum to Species) of bacteria among the different groups (LDA score > 4, *p* < 0.05). The metagenomic function was predicted by PICRUSt2 (Phylogenetic Investigation of Communities by Reconstruction of Unobserved States) based on OTU representative sequences.

### LC/MS data processing and differential metabolites identification

The methods and steps referred to the papers of other scholars (Xie et al., 2019; Wang et al., 2019a,b; Ren et al., 2022). The metabolites were extracted using a 400 μL methanol: water (4,1, v/v) solution with 0.02 mg/mL L-2-chlorophenylalanin as internal standard. After protein precipitation and centrifugation, the supernatant was carefully transferred to sample vials for LC–MS/MS analysis. A pooled quality control sample (QC) was prepared by mixing equal volumes of all samples. The QC samples were disposed and tested in the same manner as the analytic samples. The instrument platform for this LC–MS analysis is UHPLC-Q Exactive HF-X system of Thermo Fisher Scientific. After the mass spectrometry detection is completed, the raw data of LC/MS is preprocessed by Progenesis QI (Waters Corporation, Milford, United States) software. At the same time, the metabolites were searched, identified and analyzed.

### Calculation and statistical analysis

Spearman correlation analysis was generated by calculating the Spearman correlation coefficient between microbial composition (genus level) affected by time points and metabolites. The differences were defined as statistical significance at *p* < 0.05 and defined as trends at *p* < 0.10.

## Data availability statement

The datasets presented in this study can be found in online repositories. The names of the repository/repositories and accession number(s) can be found in the article/Supplementary material.

## Ethics statement

The animal study was approved by the Zhejiang University (Hangzhou, China) Institutional Animal Protection and Use Committee. The study was conducted in accordance with the local legislation and institutional requirements.

## Author contributions

MJ and HH conceived and designed the experiment. HH, MY, and WL performed the experiment and collected the samples. HH performed the microbiome and metabolome analysis, as well as wrote the manuscript. MJ, ZL, and YW revised the manuscript. All authors reviewed and approved the final manuscript.

## Funding

This research was supported by the fund from Science and Technology Projects of Zhejiang (2021C02008, CTZB-2020080127, and 2022C02043), Li Ka Shing Foundation, China Agriculture Research System (CARS-35), National Center of Technology Innovation for Pigs, National Natural Science Foundation of China (U21A20249).

## Conflict of interest

The authors declare that the research was conducted in the absence of any commercial or financial relationships that could be construed as a potential conflict of interest.

## Publisher’s note

All claims expressed in this article are solely those of the authors and do not necessarily represent those of their affiliated organizations, or those of the publisher, the editors and the reviewers. Any product that may be evaluated in this article, or claim that may be made by its manufacturer, is not guaranteed or endorsed by the publisher.

## Footnotes

^1^https://cloud.majorbio.com

^2^http://huttenhower.sph.harvard.edu/LEfSe
