# Supplementary material for: Fecal microbial and metabolic characteristics of swine from birth to market
Source: Front Microbiol. 2023 Sep 18;14:1191392. doi: 10.3389/fmicb.2023.1191392 (PMC10543884; doi:10.3389/fmicb.2023.1191392)
Supplement: Supplementary file 1 [file Data_Sheet_1.pdf]

Figure S1 The significance of phylum-level (A) genus-level (B) abundance

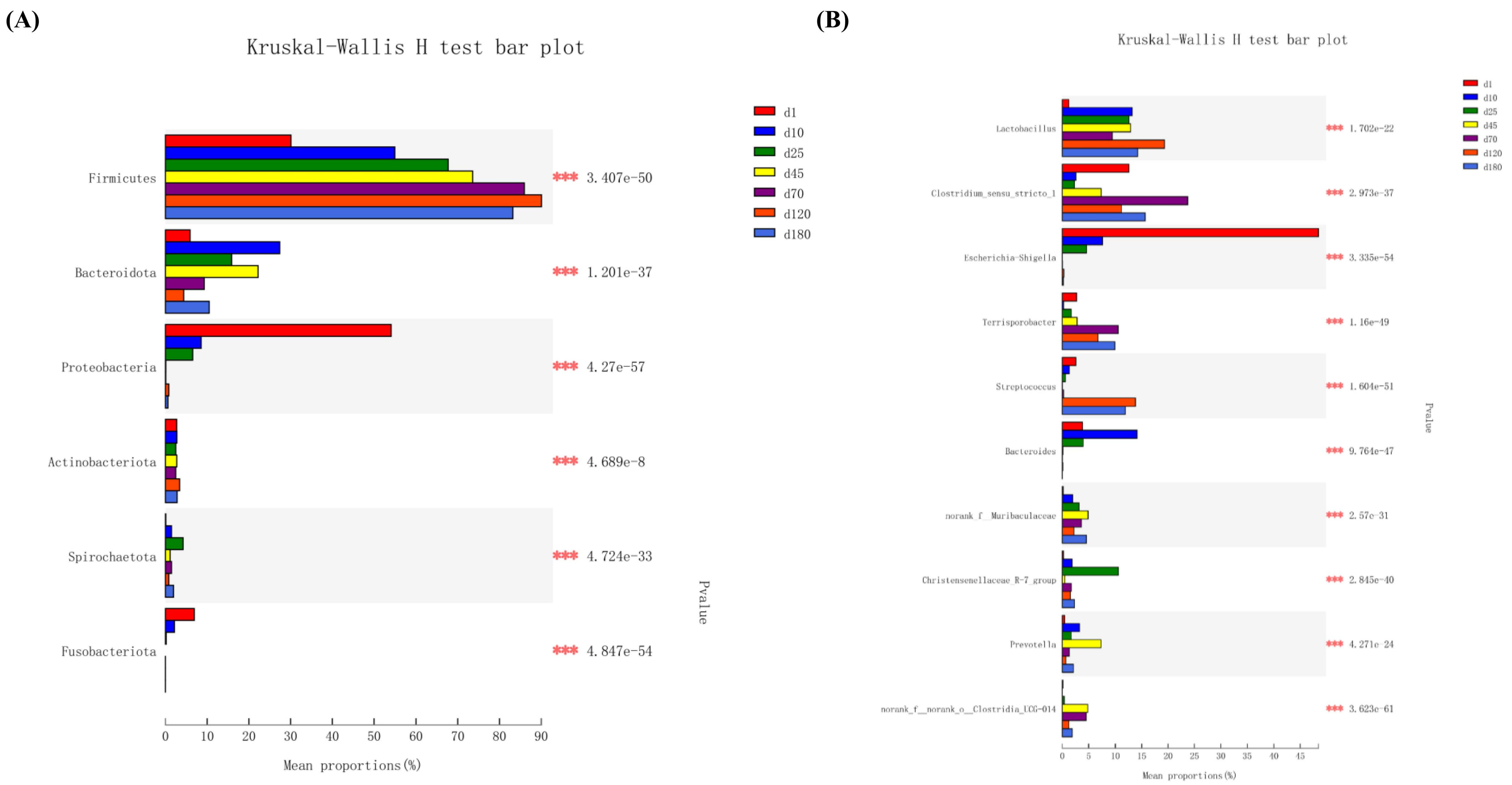

Tab.S1 The ingredient compositions for different stages of feed.

Ingredients	Day 26-Day 110	Day 111-Day 150	Day 151 -Market
Paddy, %	-	20	20
Whole-fat rice bran, %	-	5	5
Rice bran meal, %	-	3	6
Flour, %	10	5	5
Corn, %	56.1	43.85	44.8
Soybean oil, %	1.5	1.5	1.5
Soybean meal, %	10	16	12
55% concentrated protein, %	2	-	-
Puffed Soybean, %	6	-	-
Sucrose, %	1	-	-
3% whey powder, %	2	-	-
Fishmeal substitute, %	2	-	-
Montmorillonite, %	0.5	-	-
Fermented soybean meal, %	4	-	-
Fine stone powder, %	0.4	1.2	1.2
78.8% lysine , %	0.5	-	-
54.6% lysine , %	-	0.45	0.5
4% premix, %	4	4	4
Total, %	100	100	100

Tab.S2 The nutritional levels for different stages of feed.

Nutrition	Unit	Day 26-Day 110	Day 111-Day 150	Day 151 -Market
H_2O	%	11.95	12.31	12.25
CP	%	17.37	15.43	14.29
DE	Kcal/Kg	3239	3144	3107
NE	Kcal/Kg	2362	2312	2310
EE	%	5	4.51	4.54
CF	%	2.27	3.52	3.63
Ash	%	4.49	4.84	4.8
NaCl	%	0.41	0.4	0.4
Ca	%	1.09	1.05	1
P	%	0.64	0.55	0.53
Lys	%	1.3162	0.955	0.9055
Cu	mg/Kg	100.12	10.21	10.21
Fe	mg/Kg	0.34	61.97	61.98
Zn	mg/Kg	1535.78	41	41
Mn	mg/Kg	8.13	41.05	41.05
I	mg/Kg	1.4	3.1	3.1
Se	mg/Kg	0.07	0.23	4.7
V A	IU/Kg	12000	4200.21	4200.21
V D_3	IU/Kg	2400	650.03	650.03
V E	mg/Kg	68.76	26.65	76.65
V K_3	mg/Kg	7.91	1.45	1.45
V B_1	mg/Kg	4.54	6.2	6.2
V B_2	mg/Kg	8.15	1.4	1.4
V B_6	mg/Kg	3.43	2.45	2.45
V B_{12}	mg/Kg	1.74	-	-
Folic acid	mg/Kg	12.11	33.41	41.84
VH_2	mg/Kg	0.19	2.78	3.47
Niacinamide	mg/Kg	42.94	21.26	21.32
Ca pantothenate	mg/Kg	12.02	8.46	8.47
